# Low-dose ionizing radiation alleviates Aβ42-induced cell death via regulating AKT and p38 pathways in *Drosophila* Alzheimer's disease models

**DOI:** 10.1242/bio.036657

**Published:** 2019-01-22

**Authors:** Soojin Hwang, Haemin Jeong, Eun-Hee Hong, Hae Mi Joo, Kyoung Sang Cho, Seon Young Nam

**Affiliations:** 1 Low-Dose Radiation Research Team, Radiation Health Institute, Korea Hydro & Nuclear Power Co. Ltd, Seoul 01450, Korea; 2 Department of Biological Sciences, Konkuk University, Seoul 05029, Korea

**Keywords:** Amyloid-β42, Alzheimer's disease, *Drosophila*, Low-dose ionizing radiation

## Abstract

Ionizing radiation is widely used in medicine and is valuable in both the diagnosis and treatment of many diseases. However, its health effects are ambiguous. Here, we report that low-dose ionizing radiation has beneficial effects in human amyloid-β42 (Aβ42)-expressing *Drosophila* Alzheimer's disease (AD) models. Ionizing radiation at a dose of 0.05 Gy suppressed AD-like phenotypes, including developmental defects and locomotive dysfunction, but did not alter the decreased survival rates and longevity of Aβ42-expressing flies. The same dose of γ-irradiation reduced Aβ42-induced cell death in *Drosophila* AD models through downregulation of *head involution defective* (*hid*), which encodes a protein that activates caspases. However, 4 Gy of γ-irradiation increased Aβ42-induced cell death without modulating pro-apoptotic genes *grim*, *reaper* and *hid*. The AKT signaling pathway, which was suppressed in *Drosophila* AD models, was activated by either 0.05 or 4 Gy γ-irradiation. Interestingly, p38 mitogen-activated protein-kinase (MAPK) activity was inhibited by exposure to 0.05 Gy γ-irradiation but enhanced by exposure to 4 Gy in Aβ42-expressing flies. In addition, overexpression of phosphatase and tensin homolog (PTEN), a negative regulator of the AKT signaling pathway, or a null mutant of AKT strongly suppressed the beneficial effects of low-dose ionizing radiation in Aβ42-expressing flies. These results indicate that low-dose ionizing radiation suppresses Aβ42-induced cell death through regulation of the AKT and p38 MAPK signaling pathways, suggesting that low-dose ionizing radiation has hormetic effects on the pathogenesis of Aβ42-associated AD.

## Abstract (precis)

**Summary:** Low-dose ionizing radiation can reduce cell death by regulating AKT/p38 signaling pathway and improve Aβ42-induced symptoms in *Drosophila* Alzheimer's disease, suggesting that low-dose ionizing radiation may be applicable for treatment.

## INTRODUCTION

Alzheimer's disease (AD) is the most common neurodegenerative disease and is characterized by the presence of amyloid plaques, intracellular neurofibrillary tangles, progressive neuronal loss and gradual memory deterioration (Dickson, 2001; Selkoe, 2001). A major component of amyloid plaques is the aggregation of amyloid-β42 (Aβ42) protein, a pathological hallmark of AD brains (Mattson, 2004; Walsh and Selkoe, 2004). The abnormal accumulation of Aβ42, produced from amyloid precursor protein (APP), results in neuronal cell death (Yankner et al., 1990; Calhoun et al., 1998; Wei et al., 2002). Aβ42-mediated cell death in the brains of both AD patients and animal AD models has been linked to various molecular signals including activation of mitogen-activated protein kinases (MAPKs) such as p38, extracellular signal-regulated kinase (ERK) and c-Jun N-terminal kinase (JNK), as well as suppression of phosphoinositide 3-kinase (PI3K)/AKT and glycogen synthase kinase-3 (GSK-3) (Zhu et al., 2001; Pearson et al., 2006; Ma et al., 2007; Young et al., 2009; Sofola et al., 2010; Tare et al., 2011; Yin et al., 2011; Povellato et al., 2013). These pathways are being explored as potential drug targets in the treatment of AD, such as inhibition of the AKT/GSK-3β signaling pathway, for example (Van Dam and De Deyn, 2017).

To date, several drug candidates have been developed to treat AD (Mangialasche et al., 2010). N-methyl-d-aspartate (NMDA)-receptor antagonists (e.g. memantine) have been used successfully to improve AD symptoms (Mangialasche et al., 2010). Acetylcholinesterase inhibitors (e.g. Donepezil) have been effective in significantly improving cognitive impairments of AD patients (Van Dam and De Deyn, 2017). However, even with multiple drug treatments, AD patients experience progressive neuronal degeneration. The pathophysiological mechanisms underlying AD remain insufficiently characterized to identify accurate diagnostic markers and therefore potential drug targets (Van Dam and De Deyn, 2017).

Recently, positron emission tomography radiotracers to image amyloid plaques have been developed and approved for clinical use in the evaluation of suspected neurodegenerative diseases, including AD (Mallik et al., 2017). Intriguingly, low-level irradiation, in addition to its use as a diagnostic tool, is an emerging therapeutic technology and has been applied to several models of neurodegenerative disease (Song et al., 2012; Meng et al., 2013; Farfara et al., 2015; Johnstone et al., 2016). Several studies utilizing low-dose ionizing radiation in Aβ-treated mouse hippocampal neurons and the rat hippocampus suggest a potential role for low-dose ionizing radiation in AD treatment (Meng et al., 2013; Lu et al., 2017). However, *in vivo* studies examining the effects of low-dose ionizing radiation on AD outcomes are still insufficient.

*Drosophila melanogaster*, powerful genetic and cell biological model organisms, have been used in low-dose ionizing radiation research (Seong et al., 2011; Seong et al., 2012; Kim et al., 2015). In addition, *Drosophila* AD models are established, which have been useful in studying the etiology of human AD (Shulman et al., 2003; Finelli et al., 2004; Bier, 2005; Iijima-Ando and Iijima, 2010; Hong et al., 2012; Lenz et al., 2013). As *Drosophila* AD models demonstrate various easily-quantifiable phenotypes, such as eye and wing degeneration, locomotive dysfunction, shortened lifespan and developmental defects, they have been useful in the identification of AD-associated genes and pathways and in evaluating possible candidate drugs for AD treatment (Shulman et al., 2003; Blard et al., 2007; Cao et al., 2008; Rival et al., 2009; Hong et al., 2012; Park et al., 2013a,b; Xiong et al., 2013; Liu et al., 2015).

In the current study, *Drosophila* AD models were employed to investigate the effects of low-dose ionizing radiation on disease outcomes including AD-like phenotypes, such as developmental defects and locomotive dysfunction. Interestingly, low-dose ionizing radiation improved partially the AD-like phenotypes and reduced cell death by regulating AKT/p38 signaling pathway. These results suggest that low-dose ionizing radiation may exert beneficial effects on AD.

## RESULTS

### Low-dose ionizing radiation suppresses Aβ42-induced morphological defects

**Fig. 1.**
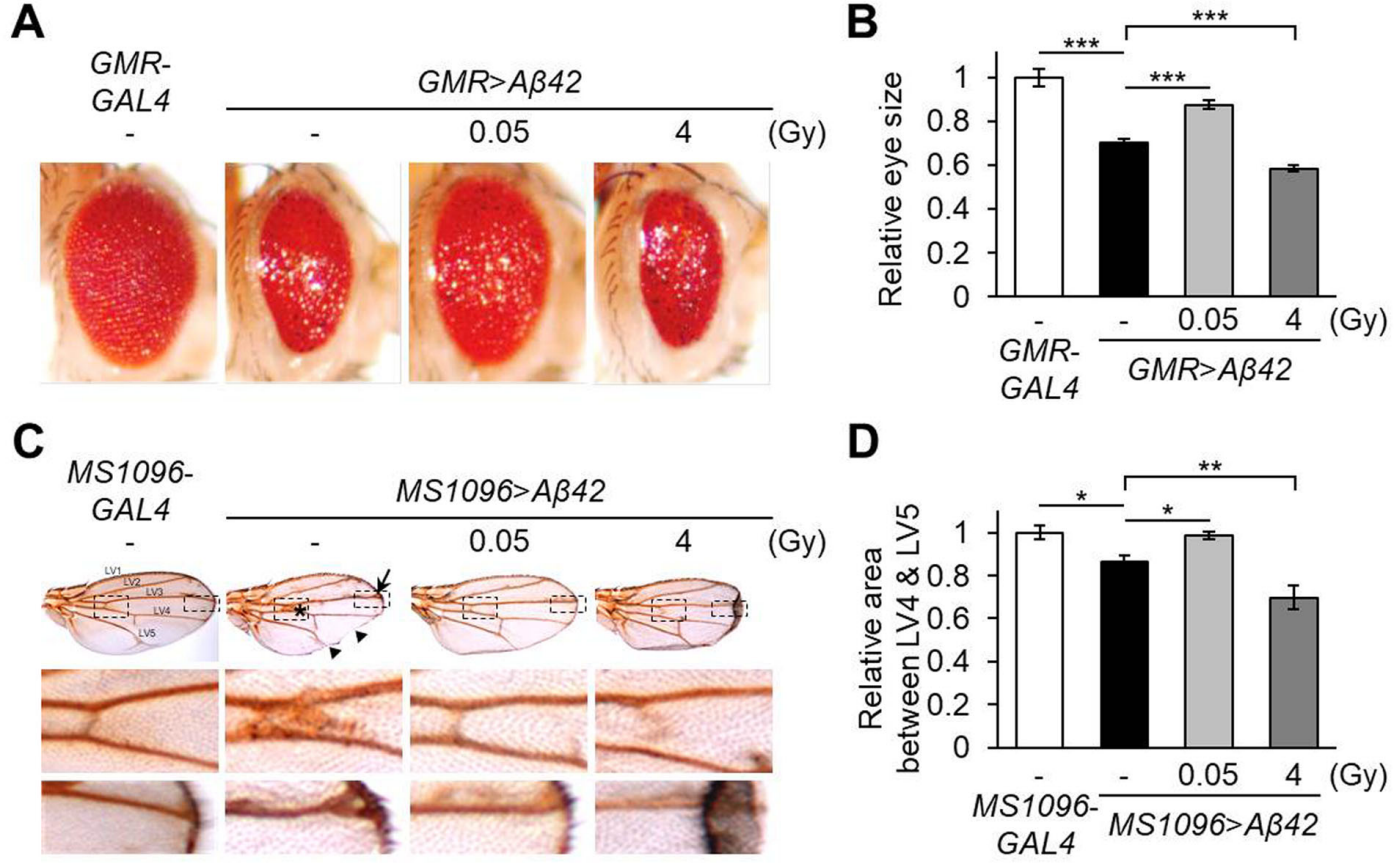
**Effects of ionizing radiation on morphological phenotypes in human Aβ42-expressing flies.** (A) The effects of low-dose (0.05 Gy) or high-dose (4 Gy) ionizing radiation on eye destruction in Aβ42-expressing flies (*GMR*>*Aβ42*) were determined. *GMR*-*GAL4* was used as a wild-type control. (B) Graph displays the relative size of eyes in each group (*n*≥6) compared to *GMR*-*GAL4* control flies. (C) Representative wing images showing the effects of γ-irradiation (0.05 Gy or 4 Gy) on the defective wing formation of Aβ42-expressing flies (*MS1096*>*Aβ42*). *MS1096*-*GAL4* was used as a wild-type control. The middle and lower images are magnified images of the two dashed boxes depicted in the upper panels. Asterisk, arrow, and triangles represent thick vein, extra vein and serration phenotypes, respectively. LV, longitudinal veins. (D) Graph shows the relative value by measuring the area between LV4 and LV5 in each wing (*n*≥6) using Image J freeware software program. The relative areas were calculated by the normalized *MS1096*-*GAL4* control flies. All data are expressed as mean±s.e.m. *P<0.05, **P<0.01, ***P<0.001. -, untreated control.

Ectopic expression of human Aβ42 in the *Drosophila* developing eye, induced by the *GMR-GAL4* driver or wing, induced by the *MS1096-GAL4* driver, results in a strong rough-eye phenotype or defective vein formations, respectively, indicating cytotoxicity (Hong et al., 2012; Park et al., 2013a,b; Liu et al., 2015). In the current study, we used these human Aβ42-expressing *Drosophila* AD models to investigate the effects of low-dose ionizing radiation. When Aβ42 was expressed in developing eyes (*GMR*>*Aβ42*), eye size was decreased to 70.1% (*P*=4.72E-05) compared to wild-type controls (*GMR*-*GAL4*) (Fig. 1A). Surprisingly, the Aβ42-induced reduction in eye size was rescued significantly to 87.5% (*P*=0.00196) with administration of low-dose γ-irradiation, 0.05 Gy, but not with high-dose, 4 Gy (Fig. 1A,B). Similarly, in the wing-specific Aβ42-expressing flies (*MS1096*>*Aβ42*), 0.05 Gy of γ-irradiation treatment improved Aβ42-induced morphological defects, including thick veins, serration phenotypes (Fig. 1C, arrows) and reduced LV4-LV5 interveinal region (Fig. 1D) compared to the wild-type controls (*MS1096*-*GAL4*). However, 4 Gy of γ-irradiation enhanced the wing shrinkage of the Aβ42-expressing flies (Fig. 1C,D). These results suggest that low-dose ionizing radiation has beneficial effects on the developmentally defective phenotypes in *Drosophila* AD models.

### Low-dose ionizing radiation ameliorates Aβ42-induced locomotive dysfunction

**Fig. 2.**
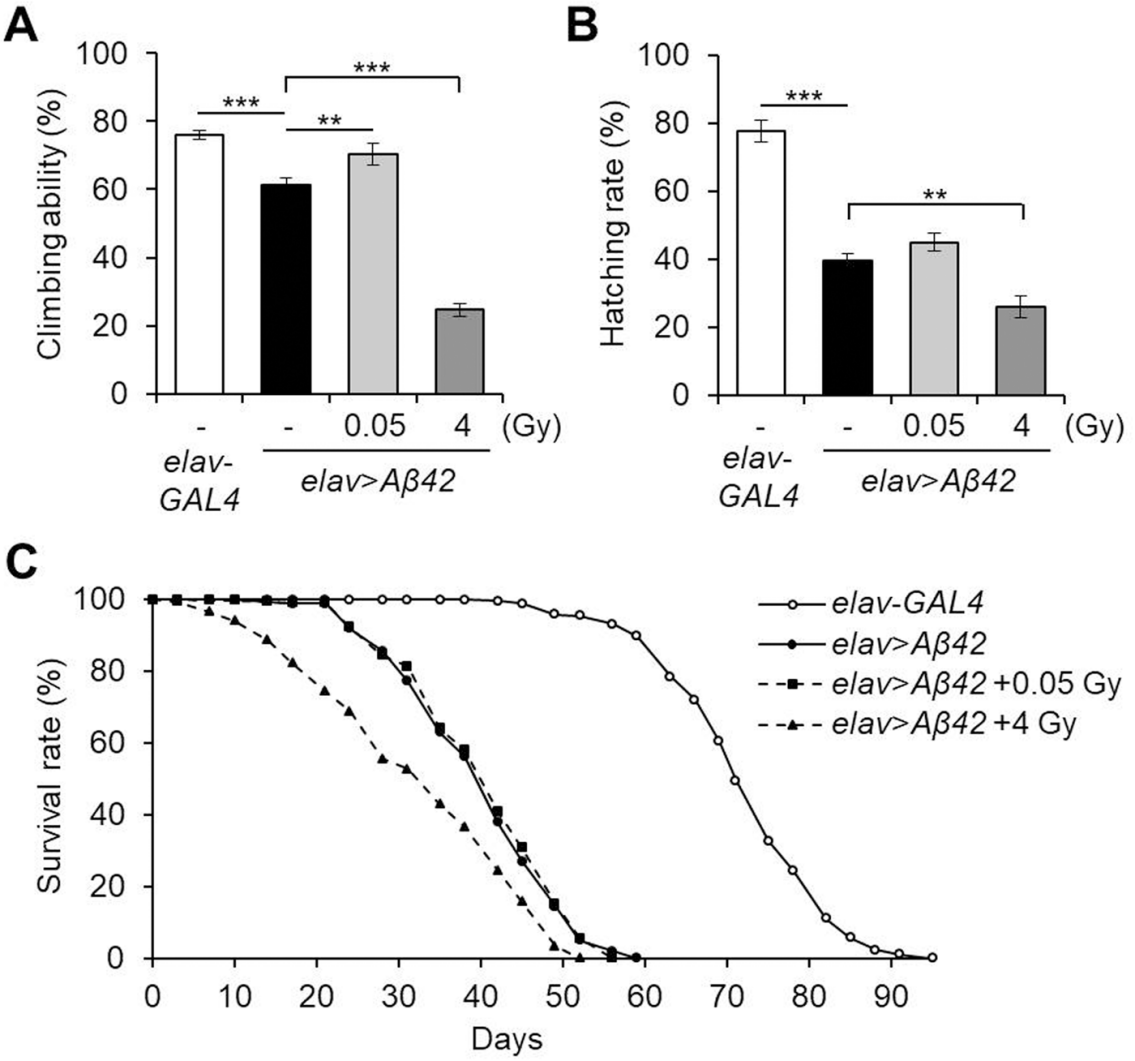
**Effects of ionizing radiation on locomotive dysfunction and survival rate of pan-neuronal Aβ42-expressing flies.** (A) The effects of low-dose (0.05 Gy) or high-dose (4 Gy) ionizing radiation on locomotive defects of pan-neuronal Aβ42-expressing flies (*elav*>*Aβ42*) were determined. The climbing ability of 3-day-old flies in each group were determined (*n*=10). (B,C) Embryonic hatching rates (*n*=5) (B) and adult survival rates (*n*≥260) (C) of Aβ42-expressing flies (*elav*>*Aβ42*) after exposure to γ-irradiation (0.05 Gy or 4 Gy). *elav*-*GAL4* was used as a wild-type control. All data are expressed as mean±s.e.m. **P<0.01, ***P<0.001. -, untreated control.

Next, to evaluate the effects of low-dose ionizing radiation on Aβ42-induced *Drosophila* neurological phenotypes, we examined the motor activity, embryonic survival rate and lifespan in γ-irradiated pan-neuronal Aβ42-expressing flies. As previously reported (Iijima et al., 2004; Hong et al., 2012; Liu et al., 2015), Aβ42 pan-neuronal expression, induced by the *elav-GAL4* driver (*elav*>*Aβ42*), decreased climbing ability, hatching rate and lifespan compared to wild-type controls (*elav*-*GAL4*) (Fig. 2). Among these phenotypes, climbing defects were significantly improved by γ-irradiation of 0.05 Gy from 61.3% to 70.3% (*P*=0.030) (Fig. 2A), but hatching rate (Fig. 2B) and lifespan (Fig. 2C) were not affected. All neuronal phenotypes, including locomotive dysfunction, decreased survival and shortened lifespan, were further deteriorated by administration of 4 Gy of γ-irradiation (Fig. 2). These results indicate that low-dose ionizing radiation, but not high-dose, can mitigate Aβ42-induced motor defects without harm to the survival and longevity of *Drosophila* in these AD models.

### Low-dose ionizing radiation improves Aβ42-induced cell death but does not alter the expression of Aβ42

**Fig. 3.**
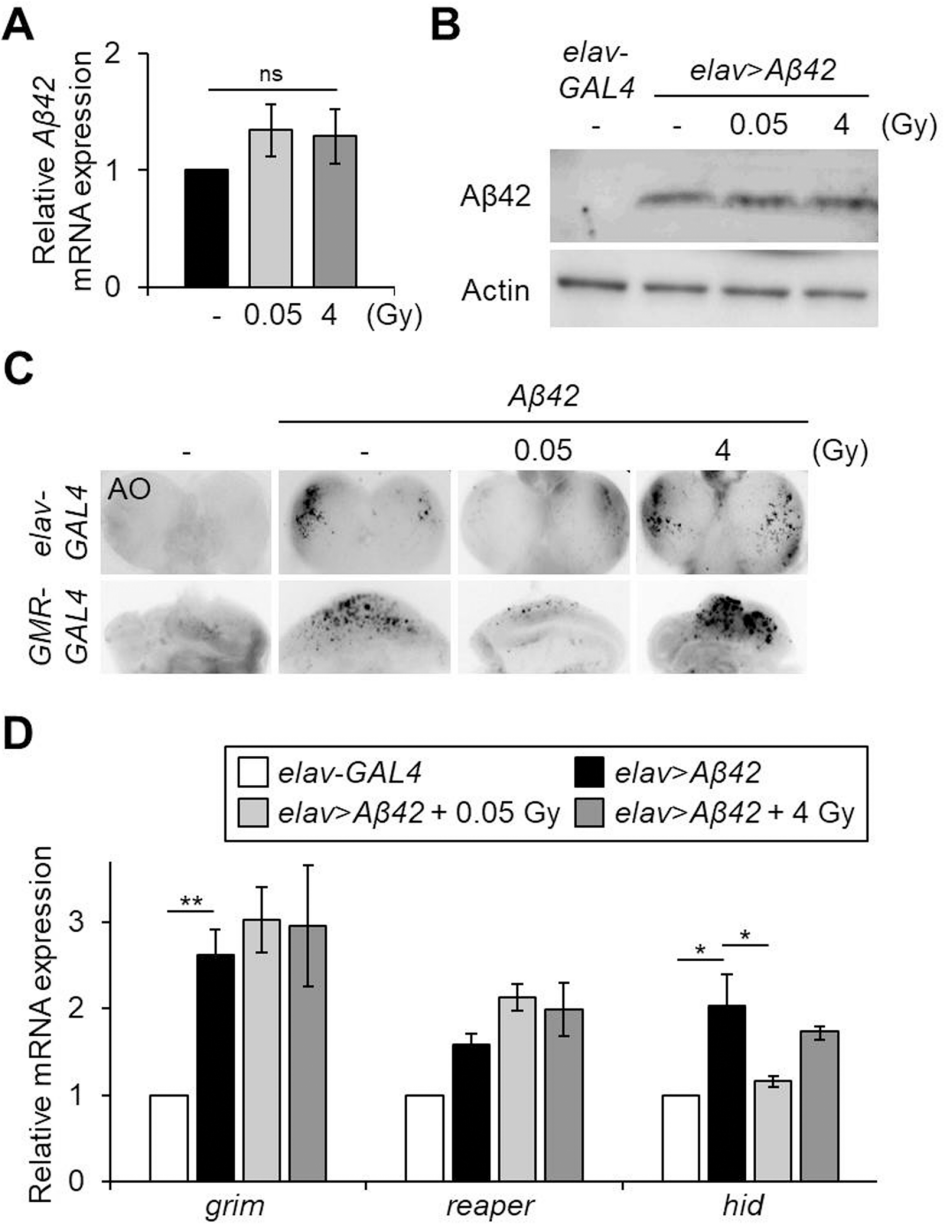
**Effects of ionizing radiation on Aβ42 protein levels, cell death and expression of pro-apoptotic genes in Aβ42-expressing flies.** (A,B) Aβ42 mRNA (A) and protein (B) expression in the heads of pan-neuronal Aβ42-expressing flies (*elav*>*Aβ42*) after exposure to low-dose (0.05 Gy) or high-dose (4 Gy) of γ-irradiation by western blot. Actin was used as an internal control. (C) AO-stained brains (upper panels) and eye discs (lower panels) of indicated larval groups. (D) Relative mRNA levels of pro-apoptotic genes *grim*, *reaper* and *hid* in the Aβ42-expressing flies (*elav*>*Aβ42*) after exposure to γ-irradiation (0.05 Gy or 4 Gy) compared to *elav*-*GAL4* control flies by qPCR (*n*=3). Data are expressed as mean±s.e.m. *P<0.05, **P<0.01. -, untreated control; ns, not significant.

As Aβ42 accumulation and neuronal cell death are important processes in the pathogenesis of AD (Wirths et al., 2004), we next examined if γ-irradiation treatment affected Aβ42 protein expression and cell death in the pan-neuronal Aβ42-expressing flies. As shown Fig. 3A,B, Aβ42 mRNA and protein levels were not altered by γ-irradiation, either 0.05 Gy or 4 Gy, suggesting that the improved or aggravated phenotypes induced by these doses of ionizing radiation, respectively, are not due to the transcription or expression of Aβ42. To investigate the effect of γ-irradiation on Aβ42-induced cell death, Acridine Orange (AO) staining was performed in the larval brain (pan-neuronal Aβ42-expressing flies driven by the *elav-GAL4* driver) and eye disc (eye-specific Aβ42-expressing flies driven by the *GMR-GAL4* driver) (Fig. 3C). As previously reported (Liu et al., 2015), Aβ42 expression in neurons or the developing eye induced a high level of cell death, while no prominent cell death was detected in the wild-type controls (Fig. 3C). Interestingly, Aβ42-induced cell death was strongly suppressed by 0.05 Gy of γ-irradiation and increased by 4 Gy of γ-irradiation (Fig. 3C). In addition, among pro-apoptotic genes, the *head involution defective* (*hid*) upregulation induced in the pan-neuronal Aβ42-expressing flies was suppressed by γ-irradiation, 0.05 Gy, but not 4 Gy (Fig. 3D). The expression levels of *grim* and *reaper* were not altered by either dose of γ-irradiation (Fig. 3D). These results indicate that the beneficial effects of low-dose ionizing radiation on Aβ42-induced phenotypes may be due to a decrease in apoptosis through regulation of *hid* expression and downstream caspase activation.

### Ionizing radiation mediates AKT and p38 MAPK signaling pathways in *Drosophila* AD models

**Fig. 4.**
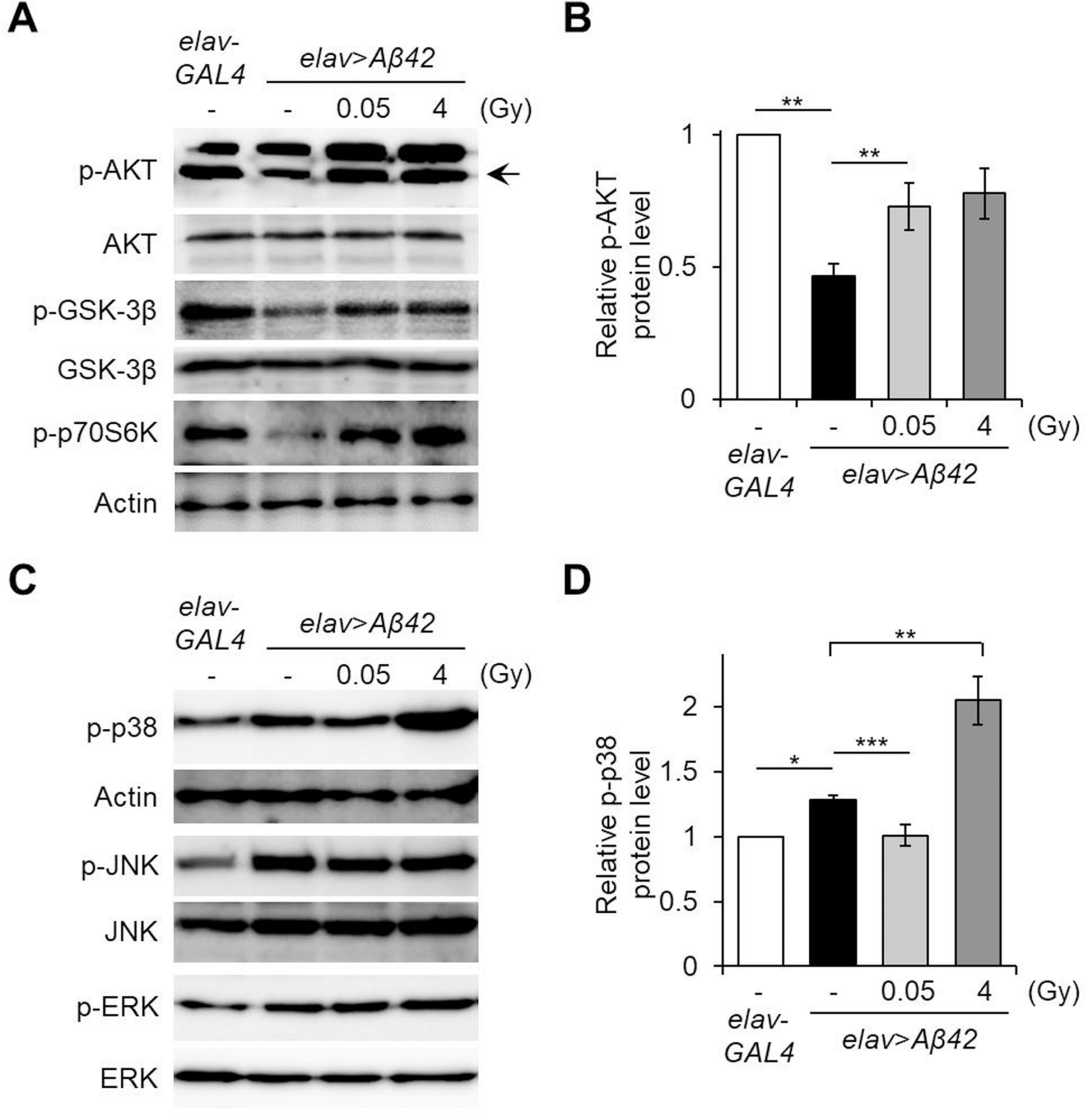
**Effects of ionizing radiation on the AKT survival pathway or MAPK pathway in Aβ42-expressing flies.** (A) The levels of phosphorylated (p)-AKT, p-GSK-3β and p-p70S6K in the heads of Aβ42-expressing flies (*elav*>*Aβ42*) after exposure to γ-irradiation (0.05 Gy or 4 Gy), compared to *elav*-*GAL4* control flies, determined by western blot. AKT, GSK-3β and actin were used as controls, respectively. (B) Graph shows the relative p-AKT levels in the heads of each group compared to *elav*-*GAL4* control flies (*n*=4). (C) The levels of p-p38, p-JNK and p-ERK in the heads of indicated groups by western blot. Actin, JNK and ERK were used as controls, respectively. (D) Graph shows the relative levels of p-p38 in the heads of each group compared to *elav*-*GAL4* control flies (*n*=5). Data are expressed as mean±s.e.m. *P<0.05, **P<0.01, ***P<0.001. -, untreated control.

Previous studies report that Aβ42 accumulation induces apoptosis through either inactivation of the AKT/GSK-3β survival signaling pathway (Magrané et al., 2005; Lee et al., 2009; Yin et al., 2011) or activation of MAPK signaling pathways such as ERK, JNK and p38 (Perry et al., 1999; Zhu et al., 2001). To investigate whether ionizing radiation influences these Aβ42-associated pathways, AKT and MAPK signaling pathway activation was assessed following treatment with ionizing radiation. The levels of downregulated phosphorylation of AKT Ser505, which corresponds with residues of Ser473 in mammalian AKT (Sarbassov et al., 2005), of phospho-GSK-3β and phospho-p70S6K in the pan-neuronal Aβ42-expressing flies (*elav*>*Aβ42*) were significantly increased by γ-irradiation treatment of 0.05 Gy and 4 Gy (Fig. 4A,B). Interestingly, the level of upregulated phospho-p38 protein in the Aβ42-expressing flies was reduced by low-dose γ-irradiation, 0.05 Gy, but further elevated by high-dose γ-irradiation, 4 Gy (Fig. 4C,D). There were no discernible differences in either phospho-JNK or phospho-ERK levels between the untreated controls and γ-irradiated Aβ42-expressing flies (Fig. 4C). These results suggest that low-dose ionizing radiation suppresses Aβ42-induced cell death through activation of the AKT survival signaling pathway and inhibition of the p38 MAPK apoptotic pathway. The harmful effects of high-dose ionizing radiation may be attributed to the hyperactivation of p38 MAPK despite activation of AKT. Therefore, balance between the AKT and p38 MAPK signaling pathways is an important factor in the cellular response to ionizing radiation.

**Fig. 5.**
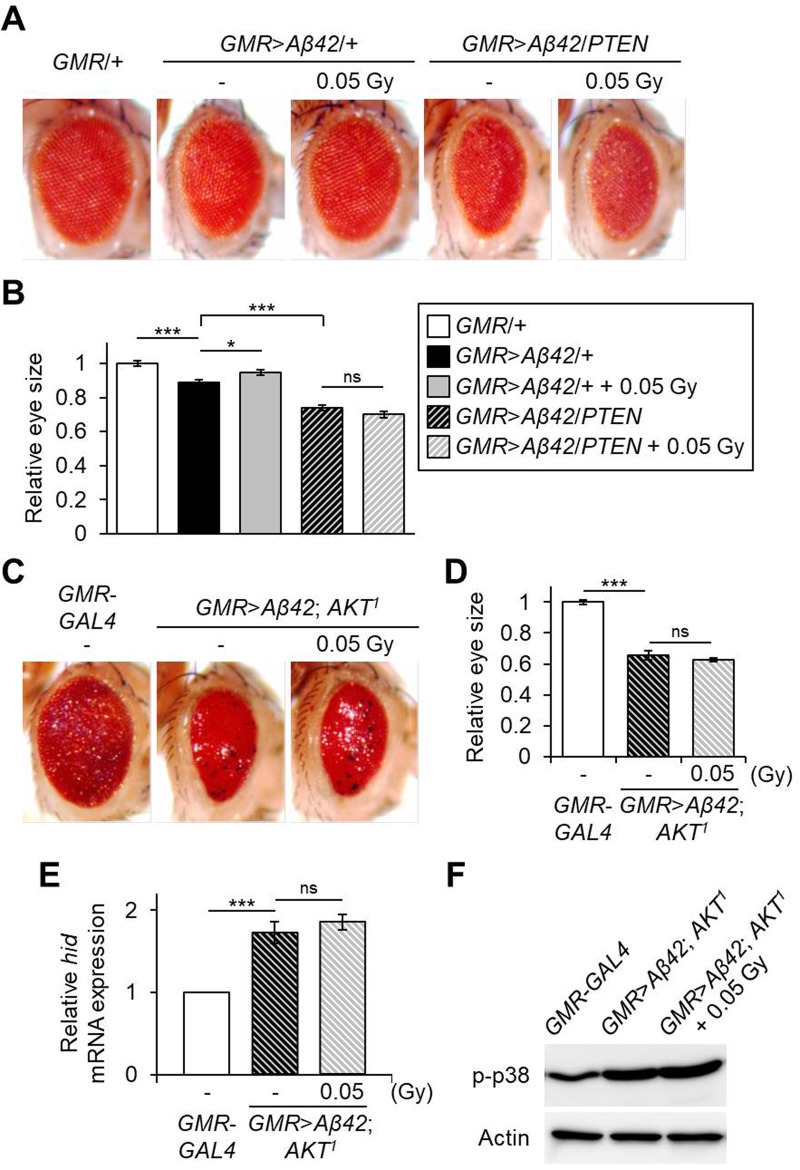
**Effects of AKT inhibition on the response to low-dose ionizing radiation in Aβ42-expressing flies.** (A) Representative eye images showing the effects of low-dose (0.05 Gy) ionizing radiation on Aβ42-expressing (*GMR*>*Aβ42*/+) or Aβ42- and PTEN-co-expressing (*GMR*>*Aβ42*/*PTEN*) flies. *GMR*/+ was used as a wild-type control. (B) Graph shows the relative size of eyes in each indicated fly group (*n*≥6) compared to *GMR*/+ control flies. (C) Representative eye images showing the effects of low-dose (0.05 Gy) ionizing radiation on AKT deficiency (*AKT^1^*) in Aβ42-expressing flies. (D) Graph shows the relative size of eyes in each indicated group (*n*≥6) compared to *GMR*-*GAL4* control flies. (E) Relative mRNA levels of *hid* in the Aβ42-expressing and AKT mutant flies (*GMR*>*Aβ42*; *AKT^1^*) after exposure to γ-irradiation of 0.05 Gy compared to *GMR*-*GAL4* control flies by qPCR (*n*=3). (F) Levels of p-p38 in the heads of indicated groups by western blot. Actin was used as an internal control. Data are expressed as mean±s.e.m. *P<0.05, ***P<0.001. -, untreated control; ns, not significant.

Finally, we investigated whether inhibition of AKT activation could suppress the beneficial effects of low-dose ionizing radiation in the Aβ42-expressing *Drosophila* AD models. To accomplish this, phosphatase and tensin homolog (PTEN), a negative regulator of the AKT signaling pathway, was overexpressed along with eye-specific Aβ42-expression. As shown in Fig. 5A,B, eye size of Aβ42- and PTEN-co-expressing flies (*GMR*>*Aβ42*/*PTEN*) was decreased to 83.1% (*P*=5.54E-06) compared to Aβ42-expressing flies (*GMR*>*Aβ42*/+). However, the treatment with γ-irradiation of 0.05 Gy did not improve eye size in the Aβ42- and PTEN-co-expressing flies (Fig. 5A,B). Also, AKT deficiency (*AKT^1^*) suppressed the positive effect of low-dose treatment in the eye-specific Aβ42-expressing flies (Fig. 5C,D). In addition, the upregulation of *hid* and p38 phosphorylation by 0.05 Gy treatment in Aβ42-expressing flies was abolished by AKT deficiency (Fig. 5E,F). Taken together, these results imply that the AKT signaling pathway is important in the response to low-dose ionizing radiation in Aβ42-associated *Drosophila* AD models.

## DISCUSSION

The effects on exposure to low-dose stresses, even though toxic at higher doses, are still debated (Sohal and Weindruch, 1996; Morimoto and Santoro, 1998; Finkel and Holbrook, 2000; Masoro, 2000; Gori and Münzel, 2012). Ionizing radiation is an important emerging therapeutic as well as diagnostic tool in medicine. However, there is controversy as to whether biological effects of low-dose ionizing radiation are beneficial or indifferent (Song et al., 2012; Meng et al., 2013; Farfara et al., 2015; Johnstone et al., 2016; Tang and Loke, 2015). Several studies on radiation hormesis support the hypothesis that low-dose ionizing radiation, generally recognized as 0.1 Gy and below, elicits beneficial cell signaling responses (Macklis and Beresford, 1991; Calabrese and Baldwin, 2000). For example, low-dose ionizing radiation stimulates various cell survival-related biological responses including DNA repair and the immune system (Gori and Münzel, 2012). However, research on the effects of low-dose ionizing radiation have been confined to *in vitro* studies, thus *in vivo* evidence is currently insufficient.

To verify the radiation hormetic effects, *Drosophila* is an ideal model system for studying the biological response to ionizing radiation (Landis et al., 2012; Moskalev et al., 2015). We previously reported that low-dose ionizing radiation enhances locomotive behavior and extends lifespan in wild-type *Drosophila* (Seong et al., 2011; Kim et al., 2015). In the present study, we confirmed the effects on low-dose ionizing radiation in human Aβ42-expressing *Drosophila* AD models. Our results demonstrated that low-dose γ-irradiation, 0.05 Gy, rescued AD-like phenotypes, including morphological defects, motor dysfunction and cell death, without compromising survival rates, embryonic hatching rates or adult lifespan. Similarly, several studies using mouse models showed that ionizing radiation is a potential therapeutic in AD (Marples et al., 2016). Opposing arguments exist that claim that low-dose ionizing radiation is actually a potential risk factor for AD. However, there are no reports of pathological or genetic data associating exposure to low-dose ionizing radiation with increased AD to date (Lowe et al., 2009). Recently, a case study reported improvements in symptoms of an AD patient after radiation exposure (Cuttler et al., 2016). Our data support the hypothesis that low-dose ionizing radiation produces beneficial effects, stimulating the activation of survival mechanisms that protect against AD.

Several recent reports suggest that cell protection-associated proteins, such as the serine/threonine kinase AKT, are associated with the molecular response to ionizing radiation exposure (Liang et al., 2016; Zhang et al., 2016). We have also reported that low-dose ionizing radiation alleviates apoptosis through the AKT and MAPK pathways (Kim et al., 2007; Park et al., 2009; Park et al., 2013a,b; Park et al., 2015). In addition, upregulation of the AKT/GSK3 signaling pathway attenuates Aβ42-induced apoptosis (Lee et al., 2009; Yin et al., 2011). As there is a pronounced decrease in AKT/GSK-3β signaling pathway activation in AD models (Magrané et al., 2005; Povellato et al., 2013), we hypothesized that low-dose ionizing radiation modulates cell death through the AKT survival signaling pathway in Aβ42-expressing AD models. Indeed, AKT, GSK-3β and p70S6K, which are suppressed in Aβ42-expressing flies, were increased and Aβ42-induced cell death was markedly reduced by γ-irradiation of 0.05 Gy. Additionally, inhibition of the AKT signaling pathway strongly suppressed the positive effects of low-dose ionizing radiation in Aβ42-expressing flies. These findings suggest that the AKT survival pathway mediates ionizing radiation-induced effects in Aβ42-expressing AD models. Low-dose ionizing radiation protects flies against Aβ42-induced cell death, at least in part, through activation of the AKT/GSK-3β/p70S6K signaling pathway.

We also demonstrated that p38 phosphorylation in Aβ42-expressing flies was further increased by high-dose γ-irradiation (4 Gy), as opposed to the suppression seen with low-dose γ-irradiation (0.05 Gy). Hyperactivation of p38 MAPK in AD models has been shown to result in apoptosis (Zhu et al., 2002; Ashabi et al., 2013; Xue et al., 2014). Consistent with this, our studies indicated that γ-irradiation of 4 Gy induced strong cell death, potentially resulting from the upregulation of p38 MAPK, despite activation of AKT signaling.

**Fig. 6.**
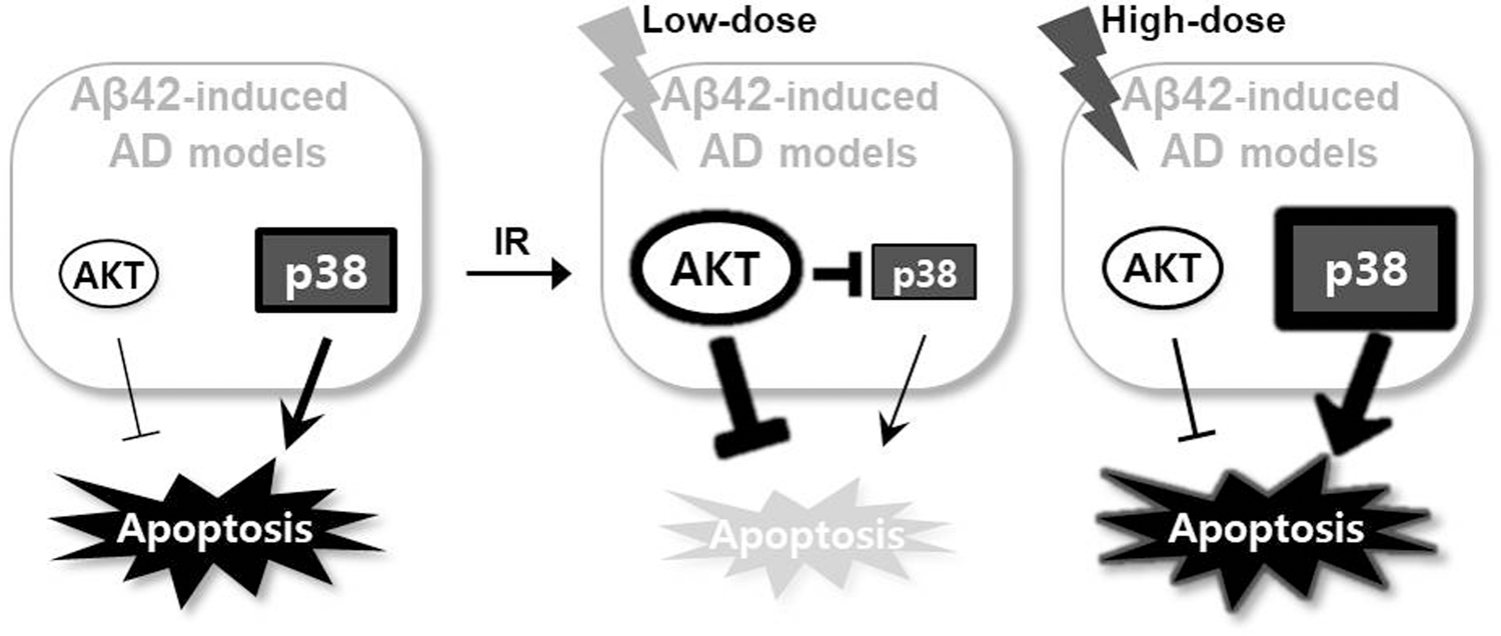
**Schematic representation of the cellular response to ionizing radiation in Aβ42-induced AD models.** Balancing between AKT and p38 pathway activation controls cellular responses to low- or high-dose ionizing radiation. Low-dose ionizing radiation induces beneficial effects against Aβ42-induced apoptosis through activation of AKT signaling and suppression of the p38 pathway in Aβ42-associated AD models.

These findings in the *Drosophila* AD models characterize the biological response to ionizing radiation treatment and a proposed model is illustrated in Fig. 6. In Aβ42-associated AD models, Aβ42 accumulation induces cell death via AKT inhibition and p38 activation. Low-dose ionizing radiation inhibits cell death in the Aβ42-induced AD models. This protection results from activation of the AKT survival signaling pathway, inhibiting cell death, and suppression of p38 activation. However, high-dose ionizing radiation, despite activation of AKT signaling, induces hyper-activated p38 leading to increased cell death. This regulation of AKT activation might play an important role in the beneficial effects of low-dose ionizing radiation on AD model outcomes. Further studies are necessary to dissect ionizing radiation-induced regulation of AKT and p38 MAPK signaling pathways and the regulatory mechanisms involved in the physiological protection against AD.

## MATERIALS AND METHODS

### Drosophila strains

*Glass multimer reporter* (*GMR*)-*GAL4* (eye driver), *embryonic lethal abnormal vision* (*elav*)-*GAL4* (pan-neuronal driver), *UAS*-*Aβ42* and *UAS*-*PTEN* were obtained from the Bloomington *Drosophila* Stock Center (stock numbers 9146, 8760, 33770 and 8549, respectively; Bloomington, IN, USA). *MS1096*-*GAL4* was generously provided by Dr M. Freeman (MRC Laboratory of Molecular Biology, Cambridge, UK) and is listed in Flybase. *AKT^1^* was obtained from Dr A. S. Manoukian (University of Toronto, Canada) (Staveley et al., 1998). All fly strains were maintained at 25°C and 60% humidity.

### γ-irradiation

γ-irradiation exposures were conducted as previously described (Kim et al., 2015), with some modification. Briefly, 0–6 h embryos were collected and immediately exposed to low-dose (0.05 Gy) and high-dose (4 Gy) ionizing radiation at a dose rate of 0.0159 Gy/s using a ^137^Cs γ-irradiator (Best Theratronics Ltd., Ottawa, ON, Canada). Both γ-irradiated embryos and non-irradiated control embryos were maintained in the same incubator at 25°C and 60% humidity.

### Analysis of *Drosophila* eyes and wings

External eye and wing morphologies were observed under dissecting microscopy (Carl Zeiss, Jena, Germany). To observe the wing vein, wings were isolated from the flies’ bodies by cutting the proximal portion. Wings were mounted in Gary's Magic Mountant solution (1.5 g Canada balsam in 1 ml methyl salicylate) on a slide glass and then it was coverslipped as previously described (Hwang et al., 2010). The size of each eye and the scores or area between longitudinal vein 4 and 5 in each wing were gauged with six or more flies per genotype using Image J freeware software program (https://imagej.nih.gov/ij) (Abramoff et al., 2004).

### Climbing assay

The climbing assay was performed as previously described (Hwang et al., 2013). Ten male flies of the indicated lines were transferred to an empty vial and incubated for 1 h at room temperature for environmental acclimation. After tapping the flies down to the bottom, the number of flies that climbed to the top of the vial within 4 s were counted. Ten trials were conducted for each group and the experiment was repeated ten times. Climbing scores (ratio of the number of flies that climbed to the top to the total number of flies, expressed as a percentage) represented the mean climbing score for ten repeated tests.

### Analysis of *Drosophila* development

Sixty embryos of each genotype were placed on grape juice agar plates. After exposure to γ-irradiation, the number of hatched larvae was counted to determine embryonic lethality. Experiments were repeated five times with 60 flies per genotype.

### AO staining

AO staining was conducted as previously described, with some modifications (Hwang et al., 2013). The brain and eye imaginal discs were dissected from stage L3 larvae in phosphate-buffered saline (PBS). In order to characterize the effects of γ-irradiation on cell death, the brain and eye discs were then incubated for 5 min in 1.6×10^−6^ M AO (Sigma-Aldrich) and briefly rinsed in PBS. The samples were subsequently observed under an Axiovert 200M fluorescence microscope (Carl Zeiss, Jena, Germany).

### Immunoblotting

For western blotting, total protein from 20 heads of 3-day-old flies was isolated from each indicated group and subjected to SDS-gel electrophoresis. Following transfer, membranes were probed with antibodies to Aβ42 (BioLegend, San Diego, CA, USA), actin (Developmental Studies Hybridoma Bank, Iowa city, IA, USA), GSK-3β (Santa Cruz Biotechnology, Dallas, TX, USA), phospho-*Drosophila* AKT (Ser505), AKT, phospho-GSK-3α/β (Ser21/9), phospho-Dp70S6K (Thr398), phospho-p38 (Thr180/Tyr182), phospho-ERK (Thr202/Tyr204), ERK, phospho-SAPK/JNK (Thr183/Tyr185) or JNK (Cell Signaling Technology, Beverly, MA, USA). Western blot analyses were conducted using standard procedures with horseradish peroxidase-conjugated secondary antibodies.

### Real-time quantitative PCR (qPCR)

For qPCR, total RNA from 20 fly heads was isolated using TRIzol reagent (Invitrogen). cDNA was synthesized using SuperScript™ II Reverse Transcriptase (Invitrogen) and qPCR was performed using SYBR Green PCR Master Mix (Applied Biosystems, Carlsbad, CA, USA) according to the manufacturer's recommended protocol. qPCR was performed using Step ONE Plus Real-time PCR system (Applied Biosystems) and the following primer pairs: *grim*, 5′-TTTGGGATTTTCTGGGAAAG-3′ and 5′-CCTCCTCATGTGTCCATACC-3′; *reaper*, 5′-ACCCAAAACCCAAACACAGT-3′ and 5′-TTGTGGCTCTGTGTCCTTGA-3′; *hid*, 5′-CAGGAGCGAAAGCAGAAAGT-3′ and 5′-TCGTGTATGTTGGCTGTTTG-3′; *actin*, 5′-TACCCCATTGAGCACGGTAT-3′ and 5′-CACACGCAGCTCATTGTAGA-3′. Quantification was performed using the ′delta-delta Ct′ method to normalize to *actin* transcript levels and control samples. Each experiment was repeated at least three times. Relative levels of mRNA were analyzed by Student's *t*-test.

### Statistical analyses

*Drosophila* eye or wing size and western blotting densitometry data were quantified with Image J freeware software program (https://imagej.nih.gov/ij) (Abramoff et al., 2004). The Student *t*-test (two-tailed) was applied for statistical significance within two groups. For comparisons of three or more groups, data was quantitatively analyzed using a one-way ANOVA by Sigma Plot 13.0 (*P<0.05, **P<0.01, ***P<0.001). For analysis of lifespan, the Kaplan–Meier estimator and the log-rank test were conducted on the pooled cumulative survival data using Online Application Survival Analysis Lifespan Assays (http://sbi.postech.ac.kr/oasis) (Yang et al., 2011).
